# Supplementary material for: Mechanically interlocked [c2]daisy chain backbone enabling advanced shape-memory polymeric materials
Source: Nat Commun. 2024 Feb 24;15:1690. doi: 10.1038/s41467-024-45980-y (PMC10894290; doi:10.1038/s41467-024-45980-y)
Supplement: Supplementary file 1 — Supplementary Information [file 41467_2024_45980_MOESM1_ESM.pdf]

# Supplementary Information for

## Mechanically Interlocked [c2]Daisy Chain Backbone Enabling Advanced Shape-Memory Polymeric Materials

Shang-Wu Zhou, [1] Danlei Zhou, [1] Ruirui Gu, [1]* Chang-Shun Ma, [1] Chengyuan Yu, [1] Da-Hui Qu[1]*

[1] Key Laboratory for Advanced Materials and Joint International Research Laboratory of Precision Chemistry and Molecular Engineering, Feringa Nobel Prize Scientist Joint Research Center, Frontiers Science Center for Materiobiology and Dynamic Chemistry, Institute of Fine Chemicals, School of Chemistry and Molecular Engineering, East China University of Science and Technology, Shanghai, 200237, P. R. China.

## 1. Supplementary Methods

### Instruments and Methods

Nuclear Magnetic Resonance (NMR) spectra were acquired on Brüker AV- 400 and AV-600 spectrometers using tetramethylsilane as the internal standard. The electronic spray ionization (ESI) mass spectra were obtained on an LCT Premier XE mass spectrometer and the electron impact (EI) mass spectra were measured on a Waters mass spectrometer. The mechanical properties of the polymer films were measured by an HY-0580 tension machine (HENGYI), and the thermal stability was measured by thermogravimetric analysis (TGA) under the nitrogen. Each sample was heated from 25 °C to 800 °C (Mettler Toledo TGA/ SDTA851, heating rate = 20 ℃ $min^{-1}$). The thermal properties were measured by the differential scanning calorimetry (DSC) under the nitrogen (TA Instruments, modulated DSC2910, 1090B). Each sample was heated from -50 °C to 120 °C with a heating rate of 10 °C $min^{-1}$, the first cycle was used to eliminate internal stresses in the material, and the second heating cycle was used to characterize the thermal properties of the material. Dynamic mechanical properties were measured by dynamic mechanical analysis (DMA) under the nitrogen (TA Instruments Q800). Fourier transform infrared spectrometer (FT-IR) (Thermo Nicolet Corporation; 7800-350/cm 0.01/cm/6700) was used to analyze the polymer samples. X-ray diffraction (XRD) patterns were obtained on a rotating anode X-ray powder diffractometer (18KW/D/max2550VB/PC) equipped with a copper target 18KW (450mA), a fully automated curved (plate) crystal graphite monochoromator and a programmed variable slit system. The EDS mappings were measured by field emission scanning electron microscope (Germany ZEISS, GeminiSEM500). High power LED lamp source (Beijing Perfectlight: PLS-LED100C) of different wavelengths (365 nm - 450 nm) with 50 W power was utilized to obtain photo-polymerized polymers.

### Methods for testing FT-IR, SAXS and XRD

The initial DCSM is a polymer film without processing; The programmed DCSM is a processed film (Processed process: the initial DCSM film is heated to 90 ℃, then the film is stretched to create a small strain about 10% strain of maximum strain. This deformation is fixed until the temperature cools to the room temperature); The recovered DCSM is actuated by heat from programmed DCSM (Processed process: when the programmed DCSM is heated above glass transition temperature, the shape of programmed DCSM will return to initial shape due to shape memory). The red dashed box is the test area for FT-IR, SAXS and XRD.

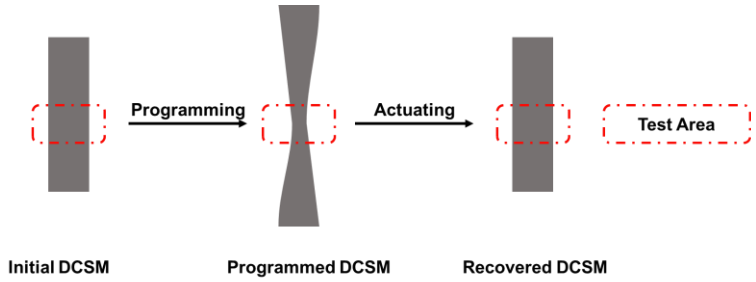


**Supplementary Fig. 1**. The polymer DCSM was characterized to be an amorphous material during the shape-memory process shown above as determined by FT-IR, SAXS, and XRD.

# Synthetic routes and characterization for DCSM-7 and DDCSM-6

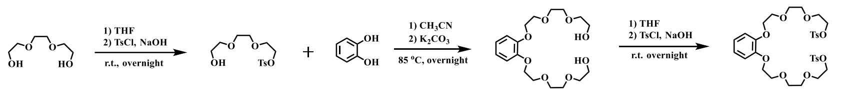


DCSM-1 DCSM-2 DCSM-3

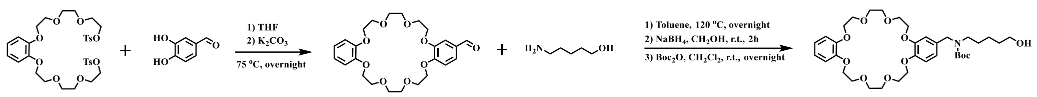


DCSM-3 DCSM-4 DCSM-5

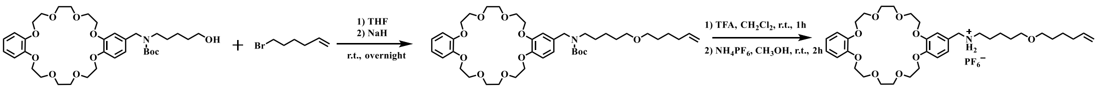


DCSM-5 DCSM-6 DCSM-7

**Supplementary Fig. 2**. Synthetic routes and chemical structures of compounds for DCSM-7. From compounds DCSM-1 to DCSM-5, these compounds had been synthesized[1-3]. DCSM-6 and DCSM-7 are new compounds.

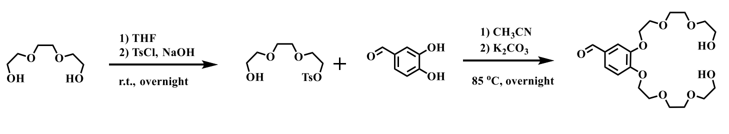


DDCSM-1 DDCSM-2

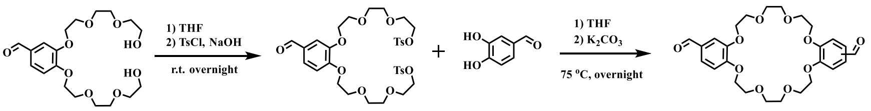


DDCSM-2 DDCSM-3 DDCSM-4

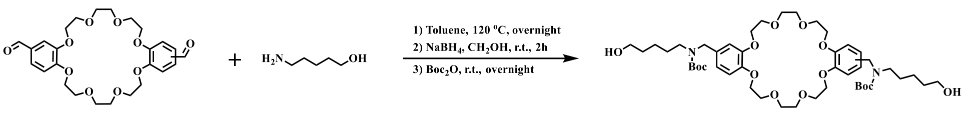


DDCSM-4 DDCSM-5

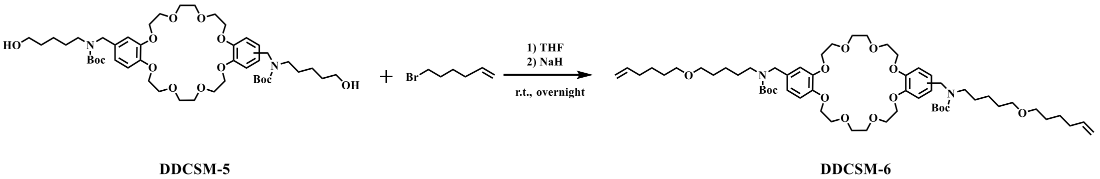


DDCSM-5 DDCSM-6

**Supplementary Fig. 3**. Synthetic routes and chemical structures of compounds for DDCSM-6. From compounds DDCSM-1 to DDCSM-4, these compounds had been synthesized[4,5]. DDCSM-5 and DDCSM-6 are new compounds.

## Synthesis of DCSM-1

Triethylene glycol (60.0 g, 400 mmol) was dissolved in 60 mL of dry THF under a

nitrogen atmosphere. The solution was cooled to 0 °C with an ice bath and NaOH (4.8 g, 120mmol) was added. Subsequently tosyl chloride (TsCl, 15.3 g, 80.0 mmol) was added in portions to the cooled reaction mixture. After complete addition, the solution was allowed to warm to room temperature and was stirred overnight. The mixture was washed with water, the aqueous fractions were re-extracted with DCM and the combined organic layers, dried over Na2SO4, filtered and the solvent was removed under vacuum. The crude oil did not need to further purify and could be used directly to be next step compound.

**Synthesis of DCSM-2**

The catechol (5.6 g, 52.0 mmol), crude DCSM-1 (31.0 g, 100.0 mmol) and $K_2CO_3$ (30.0 g, 230 mmol) in $CH_3CN$ (250 mL) was refluxed under a $N_2$ atmosphere overnight. The reaction mixture was filtrated and removed the solvent The mixture was washed with water, the aqueous fractions were re-extracted with DCM and the combined organic layers, dried over Na2SO4, filtered and the solvent was removed under vacuum. The crude oil did not need to further purify and could be used directly to be next step compound.

**Synthesis of DCSM-3**

The crude DCSM-2 (12.00 g, 32.0 mmol) were dissolved in THF (150 mL). The solution was cooled to 0 °C with an ice bath and NaOH (0.27 g, 64 mmol) was added. Subsequently tosyl chloride (TsCl, 1.2 g, 64 mmol) was added to the cooled reaction mixture. The reaction mixture was allowed to reach room temperature and stirred overnight. The reaction mixture was filtrated and removed the solvent The mixture was washed with water, the aqueous fractions were re-extracted with DCM and the combined organic layers, dried over $Na_2SO_4$, filtered and the solvent was removed under vacuum. The crude was further purified by gel chromatography with petroleum ether/ethyl acetate =1/1 as an eluent to afford DCSM-3 as a yellow oil (8.10 g, 75%). $^1H$ NMR (400 MHz, Chloroform-d) δ 7.81 - 7.76 (m, 4H), 7.32 (d, J = 8.0 Hz, 4H), 6.90 (s, 4H), 4.13 (q, J = 4.7 Hz, 8H), 3.81 (dd, J = 5.7, 4.2 Hz, 4H), 3.70 - 3.64 (m, 8H), 3.59 (dd, J = 5.6, 3.2 Hz, 4H), 2.42 (s, 6H).

**Synthesis of DCSM-4**

The DCSM-3 (8.0 g, 11.7 mmol), 3,4-dihydroxybenzaldehyde (1.6 g, 11.7 mmol) and $K_2CO_3$ (4.8 g, 35.1 mmol) were dissolved in THF (200 mL). The mixture was heated

at reflux under N2 overnight, allowed to cool to room temperature and the solids were filtered. The reaction mixture was allowed to reach room temperature and stirred overnight. The reaction mixture was filtrated and removed the solvent The mixture was washed with water, the aqueous fractions were re-extracted with DCM and the combined organic layers, dried over Na2SO4, filtered and the solvent was removed under vacuum. The crude was further purified by gel chromatography with petroleum ether/ethyl acetate = 1/2 as an eluent to afford DCSM-4 as a white powder (3.6 g, 65%). $^1$H NMR (400 MHz, Chloroform-d) δ 9.82 (s, 1H), 7.44 - 7.36 (m, 2H), 6.95 - 6.84 (m, 5H), 4.23 - 4.13 (m, 8H), 3.93 (m, 8H), 3.84 (d, J = 5.7 Hz, 8H).

**Synthesis of DCSM-5**

A solution of the DCSM-4 (3.6 g, 7.5 mmol) and the 5-amino-1-pentanol (0.93 g, 9.0 mmol) in 50 mL toluene was heated under reflux overnight. The solvent was then evaporated to give a yellow oil. The mixture was diluted with 20 mL MeOH, and then $NaBH_4$ (0.85 g, 22.5 mmol) was added portionwise at 0 °C. Stirring was maintained at room temperature for 2h. Then, methanol was evaporated, and the residue was extracted with DCM and the organic layers were combined, dried over Na2SO4 and concentrated yielding yellow oil. The yellow oil was dissolved into 50ml DCM and tert-butoxycarbonyl anhydride (6.5 g, 30.0 mmol) was added to the solutions. The reaction was stirred at room temperature overnight. The mixture was dried under reduced pressure. The crude product was purified by chromatography on a silica gel column (DCM/ MeOH= 12/ 1) to yield DCSM-5 (4.2 g, 85%) as a yellow liquid. $^1$H NMR (400 MHz, Chloroform-d) δ 6.93 - 6.70 (m, 7H), 4.38 - 4.28 (m, 2H), 4.13 (dt, J = 10.1, 4.0 Hz, 8H), 3.96 - 3.79 (m, 16H), 3.58 (t, J = 6.5 Hz, 2H), 3.13 (d, J = 39.9 Hz, 2H), 1.81 (s, 2H), 1.46 (d, J = 7.5 Hz, 14H), 1.28 (s, 2H).

**Synthesis of DCSM-6**

DCSM-5 (10.0 g, 0.015 mol) and NaH (1.8 g, 0.075 mol) were dissolved in 100 ml anhydrous tetrahydrofuran (THF) at 0°C, then the mixture stirred for 1 hour. The 6-bromo-1-hexene (12.2 g, 0.075 mol) was added to the mixture and stirred at room temperature for overnight. Then 20.0 mL water was added slowly to the mixture to quench the reaction. The mixture was dried under reduced pressure. The mixture was redissolved in 50 mL dichloromethane (DCM) and 100 mL $H_2O$, and the collected organic phase was washed with brine (3 × 10 mL). The organic layers were collected and dried with $Na_2SO_4$ and finally concentrated under reduced pressure. The crude

product was purified by chromatography on a silica gel column (DCM/ MeOH= 12/ 1) to yield DCSM-6 (8.4 g, 75%) as a yellow liquid. $^{1}$H NMR (400 MHz, $CDCl_3$) δ 6.91 - 6.83 (m, 4H), 6.81 - 6.68 (m, 3H), 5.85 - 5.73 (m, 1H), 5.02 - 4.90 (m, 2H), 4.31 (d, J = 10.0 Hz, 2H), 4.17 - 4.07 (m, 8H), 3.90 (q, J = 4.5 Hz, 8H), 3.82 (s, 8H), 3.41 - 3.32 (m, 4H), 3.10 (d, J = 35.4 Hz, 2H), 2.09 - 2.01 (m, 2H), 1.64 - 1.36 (m, 17H), 1.32 - 1.24 (m, 2H). $^{13}$C NMR (101 MHz, $CDCl_3$) δ 148.93, 148.02, 138.92, 131.87, 121.66, 121.26, 114.57, 114.23, 113.84, 113.50, 71.73, 71.32, 70.79, 70.40, 69.97, 69.52, 69.40, 6.00, 33.63, 29.55, 29.01, 28.71, 28.38, 23.61 (Supplementary Fig. 5). HRMS (ESI) (m/z): $[M + Na]^+$ calcd for $C_{41}H_{63}NO_{11}Na^+$: 768.4293, found 768.4300.

**Synthesis of DCSM-7**

DCSM-6 (8.0 g, 0.011 mol) was dissolved in 50 ml DCM, then trifluoroacetic acid (TFA, 0.055 mol, 6.3 g) was added to the mixture and stirred for 1 hour at room temperature. After the solvent was reduced under vacuum, the residue was dissolved in $CH_2Cl_2$ (50.0 mL). Then, saturated aqueous $NH_4PF_6$ solution (20.0 mL) was added, the mixture was stirred for 2 h. The mixture was dried under reduced pressure The mixture was redissolved in 50 mL dichloromethane (DCM) and 100 mL $H_2O$, and the collected organic phase was washed with brine (3 × 10 mL). After removal of the solvent, the crude product was purified by chromatography on a silica gel column ($CH_2Cl_2$/ MeOH = 20/ 1) to give DCSM-**7** (8.1 g, 92%) as a white solid. $^{1}$H NMR (400 MHz, DMSO-$d_6$) δ 8.53 (s, 2H), 7.09 (s, 1H), 7.03 - 6.97 (m, 2H), 6.97 - 6.90 (m, 2H), 6.87 (m, 2H), 5.85 - 5.73 (m, 1H), 5.03 - 4.92 (m, 2H), 4.16 - 4.00 (m, 10H), 3.78 (dd, J = 14.9, 4.2 Hz, 8H), 3.66 (d, J = 3.7 Hz, 8H), 3.35 (d, J = 2.6 Hz, 2H), 2.86 (t, J = 7.9 Hz, 2H), 2.02 (q, J = 7.1 Hz, 2H), 1.64 - 1.22 (m, 12H). $^{13}$C NMR (101 MHz, DMSO-d6) δ 148.46, 138.70, 122.96, 121.17, 115.45, 114.83, 114.04, 113.99, 113.53, 70.48, 70.44, 69.81, 69.59, 69.19, 69.07, 68.89, 68.77, 68.70, 49.87, 46.29, 32.98, 28.70, 25.26, 24.99, 22.88 (Supplementary Fig. 8). HRMS (ESI) (m/z): $[M - PF_6^-]^+$ calcd for $C_{36}H_{56}NO_9^+$: 646.3950, found 646.3954.

**Synthesis of DDCSM-5**

DDCSM-4 (9.6 g, 0.019 mol) and 5-amino-1-pentanol (4.4 g, 0.042 mol) were dissolved in 150 ml toluene and stirred under reflux overnight. After the solvent was reduced under vacuum, the residue was dissolved with $CH_3OH$ (50.0 mL). $NaBH_4$ (7.2 g, 0.19 mol) was added to the mixture by portions at 0 °C. The mixture was stirred at room temperature for 4 h; then, 20 ml water was added to the mixture slowly to quench

the reaction, Methanol was evaporated, and the residue was extracted with $CH_2Cl_2$ (3 × 20mL). The combined organic phase was washed with $H_2O$ (2 × 20mL). The organic layer was dried over $Na_2SO_4$ and concentrated under reduced pressure. The residue was dissolved in $CH_2Cl_2$ (50.0 ml), then di-tert-butyl decarbonate ($Boc_2O$, 16.5 g, 0.076 mol) was added into the mixture and stirred under at room temperature overnight. After the solvent was reduced under vacuum, the residue was extracted with $CH_2Cl_2$ (3× 20mL). The combined organic phase was washed with $H_2O$ (2 × 20mL). The organic layer was dried over $Na_2SO_4$ and concentrated under reduced pressure. The crude product was purified by chromatography on a silica gel column ($CH_2Cl_2$/ MeOH = 50/ 1) to give DDCSM-5 (13.3 g, 78%) as a yellow liquid. $^1H$ NMR (400 MHz, $CDCl_3$) δ 6.81 - 6.67 (m, 6H), 4.68 - 4.59 (m, 0H), 4.29 (d, J = 9.8 Hz, 4H), 4.10 (q, J = 4.3, 3.9 Hz, 8H), 3.91 - 3.85 (m, 8H), 3.80 (s, 8H), 3.62 - 3.51 (m, 5H), 3.21 - 2.99 (m, 5H), 2.25 - 1.95 (m, 3H), 1.43 (d, J = 12.3 Hz, 24H), 1.26 (s, 4H). $^{13}C$ NMR (101 MHz, $CDCl_3$) δ 147.86, 146.93, 130.77, 119.04, 112.64, 111.92, 70.24, 70.21, 68.87, 68.84, 68.43, 68.28, 61.51, 49.12, 48.63, 45.25, 31.25, 27.46, 27.40, 21.89. HRMS (ESI) (m/z): $[M + Na]^+$ calcd for $C_{47}H_{78}N_2O_{14}Na^+$: 901.5032, found 901.5040.

**Synthesis of DDCSM-6**

The DDCSM-5 (10.0 g, 0.011 mol) and NaH (2.1 g, 0.088 mol) were dissolved in 100 ml anhydrous tetrahydrofuran (THF) at 0°C, then the mixture stirred for 1 hour. The 6-bromo-1-hexene (14.3 g, 0.088 mol) was added to the mixture and stirred at room temperature for overnight. Then 20.0 mL water was added slowly to the mixture to quench the reaction. The mixture was dried under reduced pressure. The mixture was redissolved in 50 mL dichloromethane (DCM) and 100 mL $H_2O$, and the collected organic phase was washed with brine (3 × 10 mL). The organic layers were collected and dried with $Na_2SO_4$ and finally concentrated under reduced pressure. The crude product was purified by chromatography on a silica gel column (DCM/ MeOH = 12/ 1) to yield the DCSM-6 (8.4 g, 75%) as a yellow liquid. $^1H$ NMR (400 MHz, $CDCl_3$) δ 6.80 - 6.70 (m, 6H), 5.87 - 5.71 (m, 2H), 5.02 - 4.90 (m, 4H), 4.31 (d, J = 9.7 Hz, 4H), 4.11 (dd, J = 5.0, 3.2 Hz, 8H), 3.90 (t, J = 4.2 Hz, 8H), 3.82 (s, 8H), 3.40 - 3.33 (m, 8H), 3.11 (d, J = 35.3 Hz, 4H), 2.05 (q, J = 7.1 Hz, 4H), 1.59 - 1.51 (m, 8H), 1.49 - 1.40 (m, 26H), 1.27 (t, J = 7.8 Hz, 4H). $^{13}C$ NMR (101 MHz, $CDCl_3$) δ 148.93, 147.98, 138.77, 131.83, 114.50, 113.67, 71.28, 71.26, 70.73, 69.91, 69.88, 69.49, 69.33, 33.57, 29.49, 29.20, 28.48, 25.48, 23.55. HRMS (ESI) (m/z): $[M + Na]^+$ calcd for $C_{58}H_{94}N_2O_{14}Na^+$: 1065.6597, found 1065.6605.

**Preparation of CG-1, CG-2, and CG-3 polymers.**

CG-1 polymer: The 1,9-decadiene (260 mg, 1.88 mmol) was dissolved in anhydrous $CH_2Cl_2$ (1 ml) in 5 ml vial and stirred for 30 mins at room temperature. DCSM-SH (460 mg, 0.94 mmol) and photoinitiator (2,2-dimethoxy-1,2-diphenylethanone,7 mg, 0.13 mmol) dissolved in anhydrous $CH_2Cl_2$ (0.5 ml) was then added and stirred for 10 mins at room temperature. The precursor solution was then poured into a Teflon mold (Length * Width * Depth = 80 mm * 6 mm * 1 mm). The mixture was then irradiated under ultraviolet light (50 mW $cm^{-2}$) for 15 mins. The obtained film was dried under vacuum overnight at 70 °C.

CG-2 polymer: The DDCSM-6 (393 mg, 0.37 mmol) was dissolved in anhydrous $CH_2Cl_2$ (1 ml) in 5 ml vial and stirred for 30 mins at room temperature. DCSM-SH (92 mg, 0.19 mmol) and photoinitiator (2,2-dimethoxy-1,2-diphenylethanone, 5 mg, 0.019 mmol) dissolved in anhydrous $CH_2Cl_2$ (0.5 ml) was then added and stirred for 10 mins at room temperature. The precursor solution was then poured into a Teflon mold (Length * Width * Depth = 80 mm * 6 mm * 1 mm). The mixture was then irradiated under ultraviolet light (50 mW $cm^{-2}$) for 15 mins. The obtained film was dried under vacuum overnight at 70 °C.

CG-3 polymer: The DCSM-**7** (300 mg, 0.38 mmol) was dissolved in anhydrous $CH_2Cl_2$ (4 ml) in 5 ml vial and stirred for 30 mins at room temperature. The DCSM-SH (46 mg, 0.094 mmol) and photoinitiator (2,2-dimethoxy-1,2-diphenylethanone, 5 mg, 0.019 mmol) were added to the mixture and stirred for 10 mins. The precursor solution was then poured into a teflon mold (Length * Width * Depth = 80 mm * 6 mm * 1 mm). The mixture was then irradiated under ultraviolet light (50 mW $cm^{-2}$) for 15 mins. The obtained solution was added with triethylamine(480mg) and stirred for 30 mins. The mixed solution is viscous with precipitated salt particles. After filtration, we spread the solution on a substrate for solvent evaporation to prepare the desired polymers. The materials were obtained after evaporation of the solvent, and dried under vacuum overnight at 70 °C.

## 2. Supplementary Figures

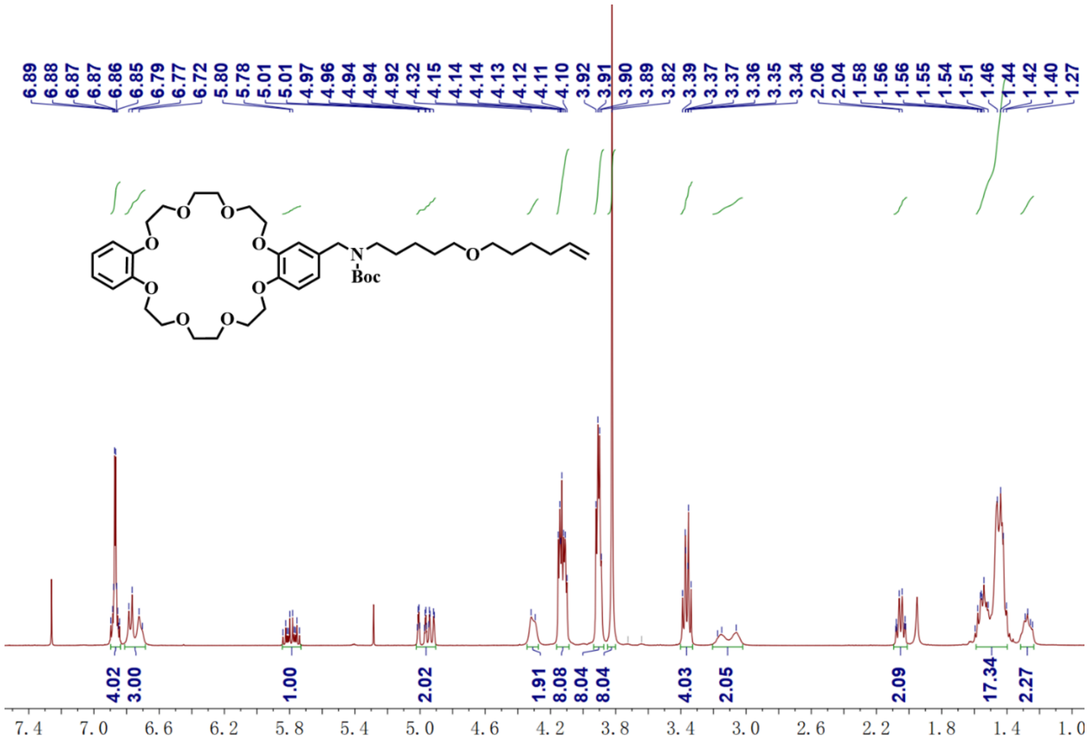


**Supplementary Fig. 4**. The $^{1}H$ NMR spectrum of DCSM-6 (400MHz, 298 K, $CDCl_3$).

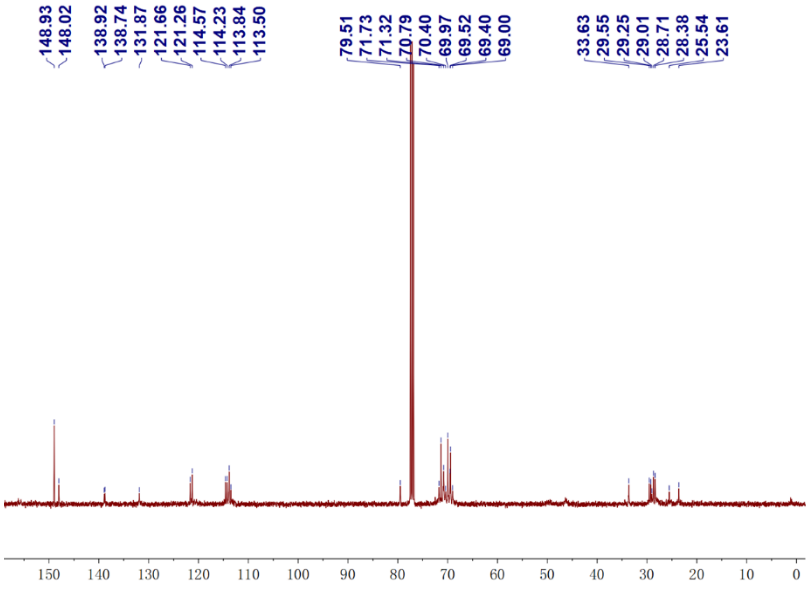


**Supplementary Fig. 5**. The $^{13}C$ NMR spectrum of DCSM-6 (101MHz, 298 K, $CDCl_3$).

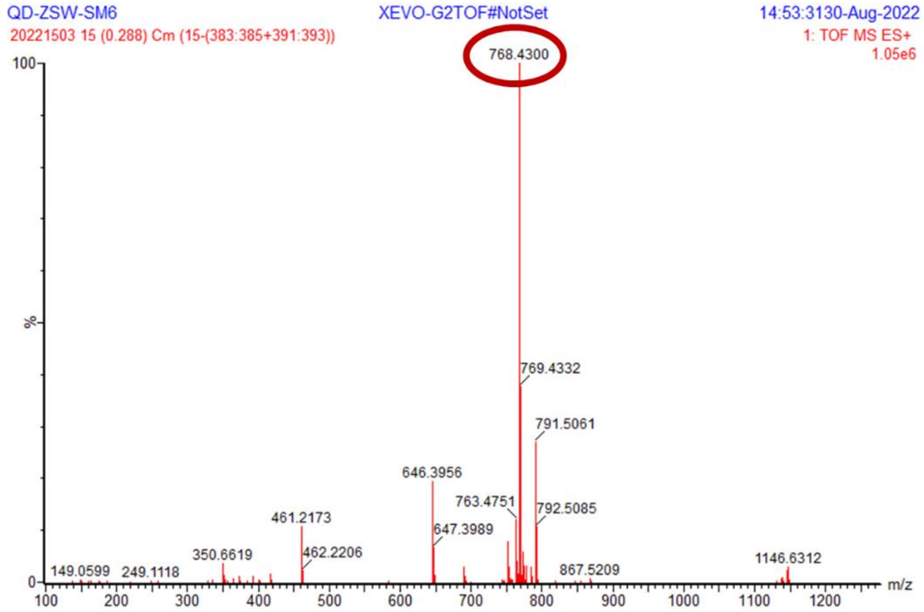


**Supplementary Fig. 6**. The ESI-mass spectrum of DCSM-6 ([M + Na$^{+}$] $^{+}$: 768.4300).

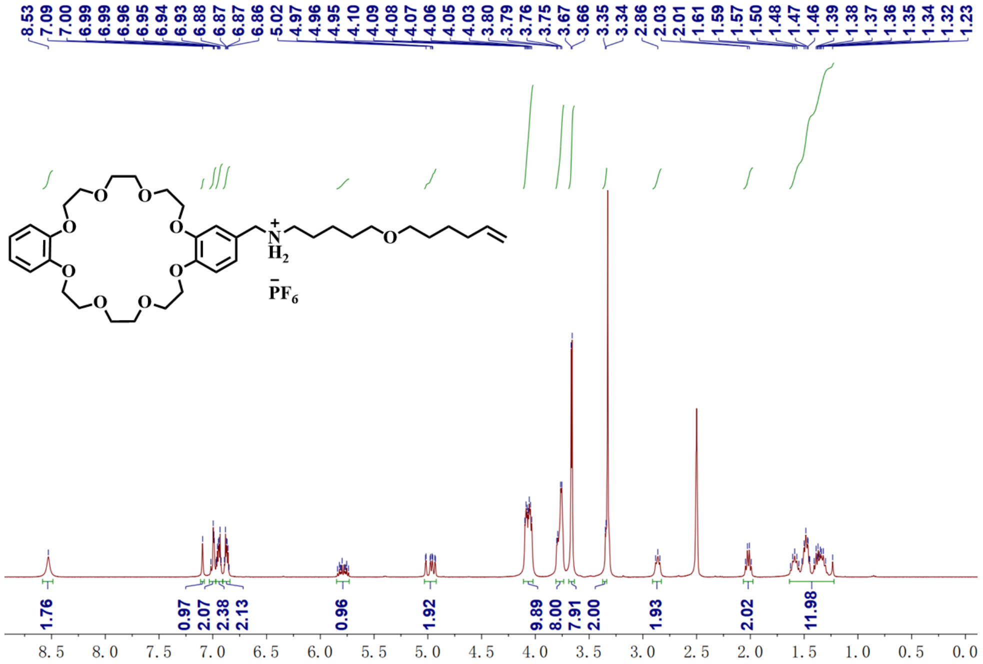

**Supplementary Fig. 7**. The $^{1}$H NMR spectrum of DCSM-7 (400MHz, 298 K, DMSO-d$_6$).

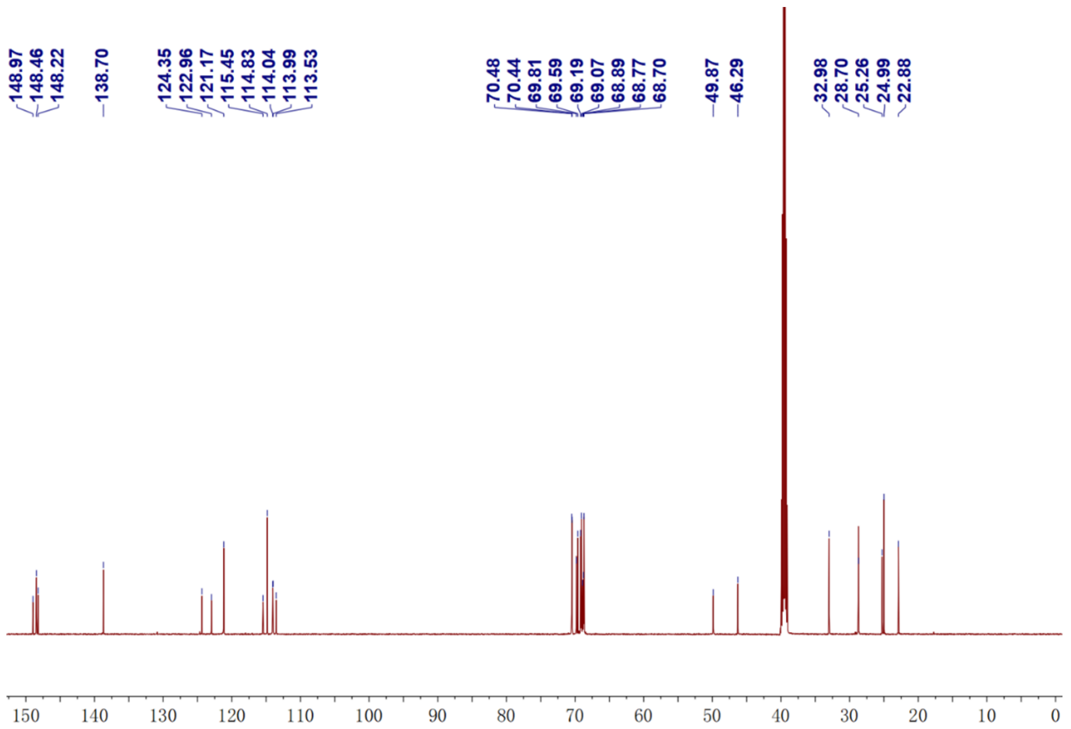


**Supplementary Fig. 8**. The $^{13}C$ NMR spectrum of DCSM-7 (101MHz, 298 K, DMSO-$d_6$).

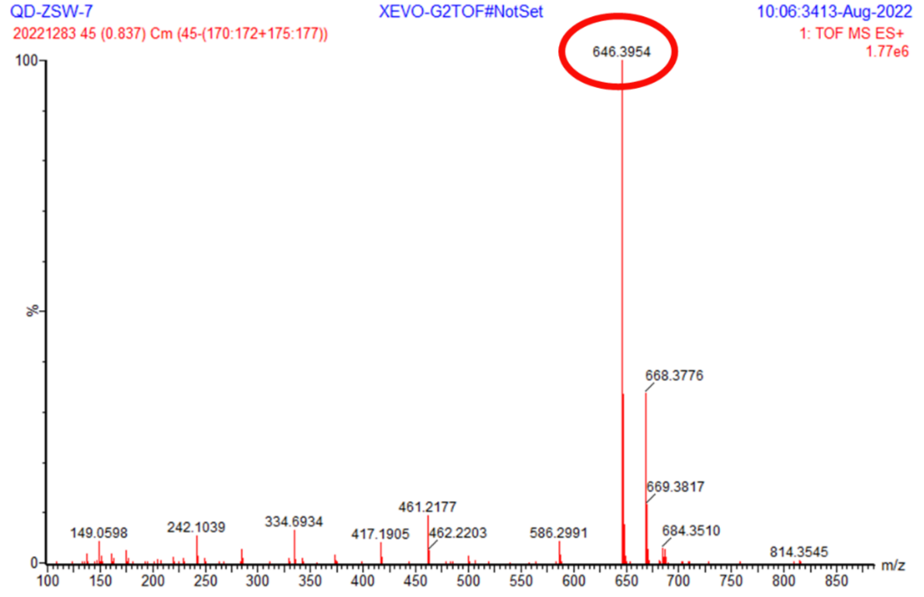


**Supplementary Fig. 9**. The ESI-mass spectrum of DCSM-7 ($[M - PF_6^-]^+$: 646.3954).

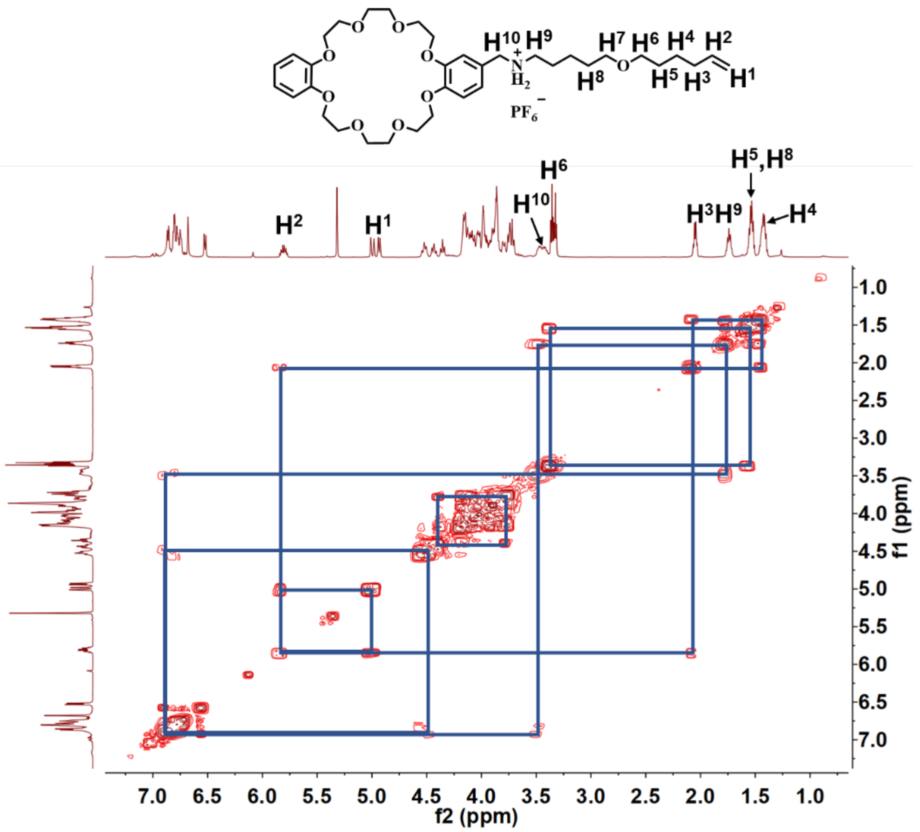


**Supplementary Fig. 10**. The correlation spectroscopy (COSY) NMR ($CD_2Cl_2$, room temperature, 600 MHz) spectrum of monomer DCSM-7. Protocol: The monomer DCSM-7 dissolved in 0.6 ml $CD_2Cl_2$ and stirred for 30 min in NMR tube, then the NMR tube was test for correlation spectroscopy (COSY) NMR.

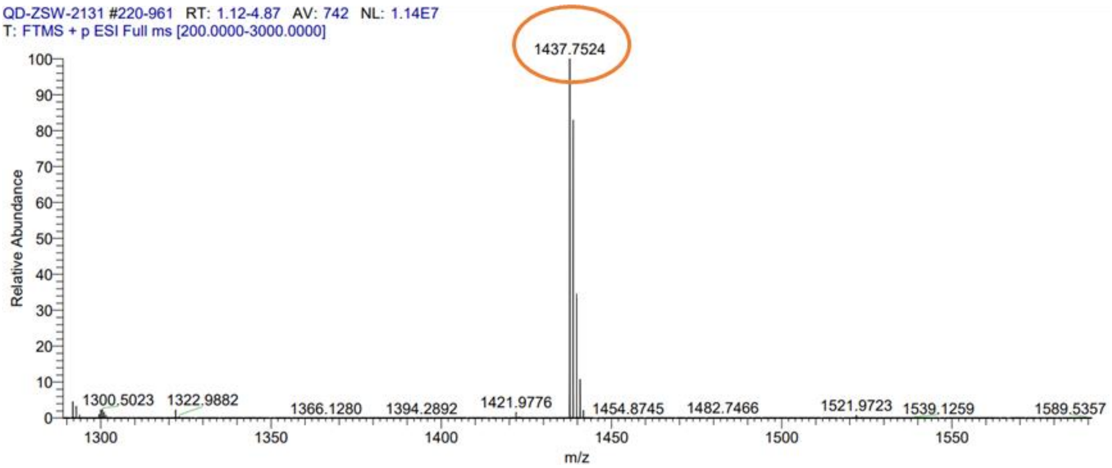


**Supplementary Fig. 11** The ESI-mass spectrum of assembled [c2]daisy chain ($[M - PF_6^-]^+$: 1437.7524). Protocol: The monomer DCSM-7 dissolved in 0.6 ml $CH_2Cl_2$ and stirred for 30 min in NMR tube, then the NMR tube was test for ESI-mass spectrum.

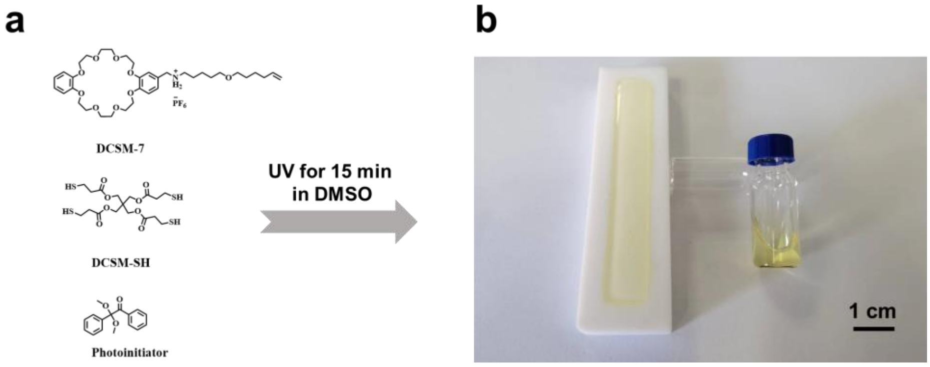


**Supplementary Fig. 12**. **a**. The preparation progress for DCSM polymerization in dimethylsulfoxide (DMSO, polar solvent). **b**. The picture for DCSM polymerization result. The DCSM-**7** could not assemble in DMSO, so it could not prepare the film but viscous yellow liquid after photoinitiation. Protocol: The DCSM-7 (300 mg) was dissolved in DMSO (1 ml) in 5 ml vial and stirred for 30 mins at room temperature. The DCSM-SH (46 mg) and photoinitiator (2,2-dimethoxy-1,2-diphenylethanone, 5 mg) were dissolved in DMSO (0.5 ml) then added to the mixture and stirred for 10 mins. The precursor solution was then poured into a teflon mold (Length * Width * Depth = 80 mm * 6 mm * 1 mm). The mixture was then irradiated under ultraviolet light (50 mW/cm$^2$) for 15 mins.

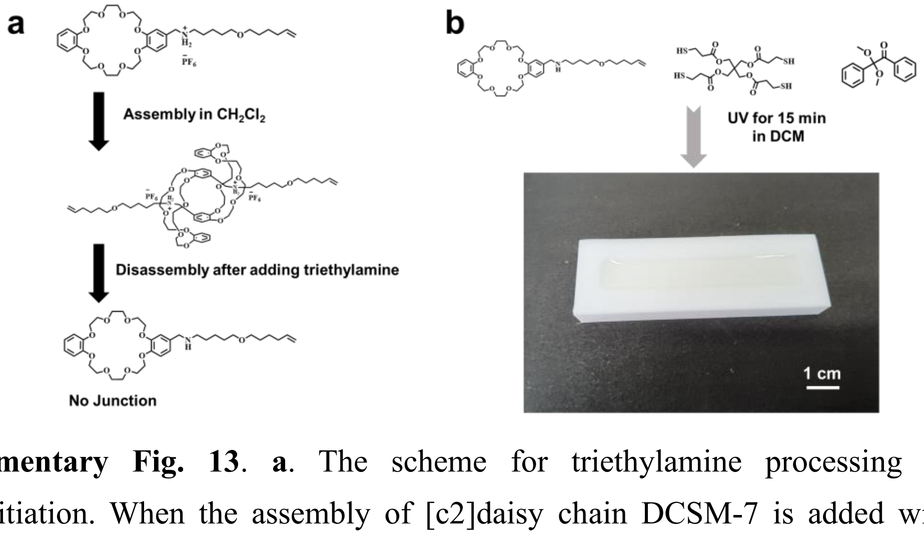


**Supplementary Fig. 13**. **a**. The scheme for triethylamine processing before photoinitiation. When the assembly of [c2]daisy chain DCSM-7 is added with the triethylamine, the assembly of [c2]daisy chain DCSM-7 will disassemble. **b**. The picture for triethylamine processing after photoinitiation. The junction of assembly

DCSM-7 disassembles after adding triethylamine in $CH_2Cl_2$, so it could not prepare the film but viscous liquid after photoinitiation. Protocol: The DCSM-7 (300 mg) was dissolved in DCM (1 ml) in 5 ml vial and stirred for 30 mins at room temperature. The DCSM-SH (46 mg) and photoinitiator (2,2-dimethoxy-1,2-diphenylethanone, 5 mg) were dissolved in DMSO (0.5 ml) then added to the mixture and stirred for 10 mins. The precursor solution was added with the triethylamine (94 mg) and stirred for 10 mins, then poured into a teflon mold (Length * Width * Depth = 80 mm * 6 mm * 1 mm). The mixture was then irradiated under ultraviolet light (50 mW/$cm^2$) for 15 mins.

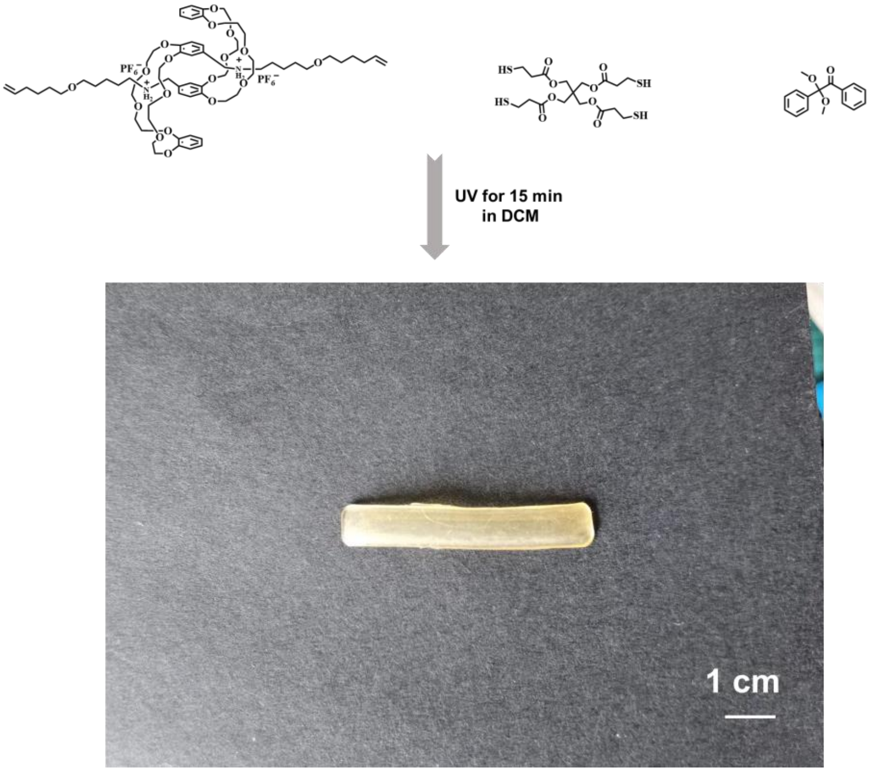


**Supplementary Fig. 14**. The preparation progress and picture of polymerization result for DCSM polymer film.

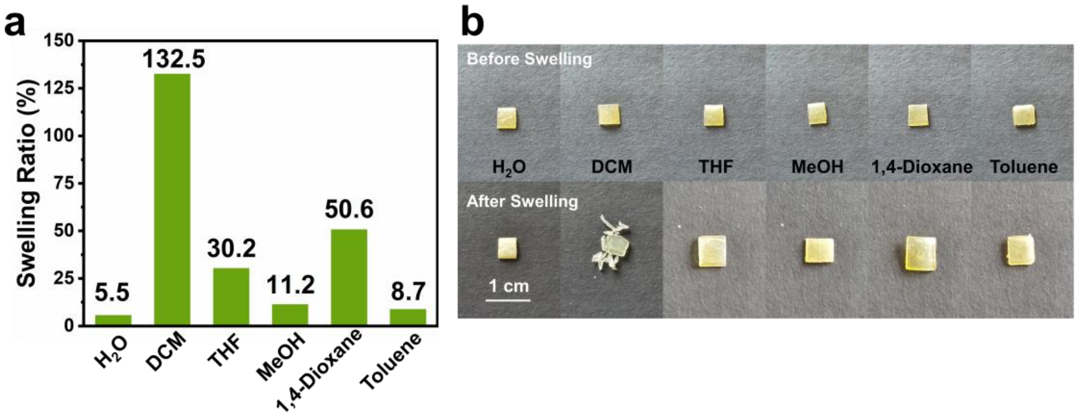


**Supplementary Fig. 15**. a. The swelling ratio for DCSM in different solvents for 3

hours. b. The before and after swelling picture for DCSM in different solvents.

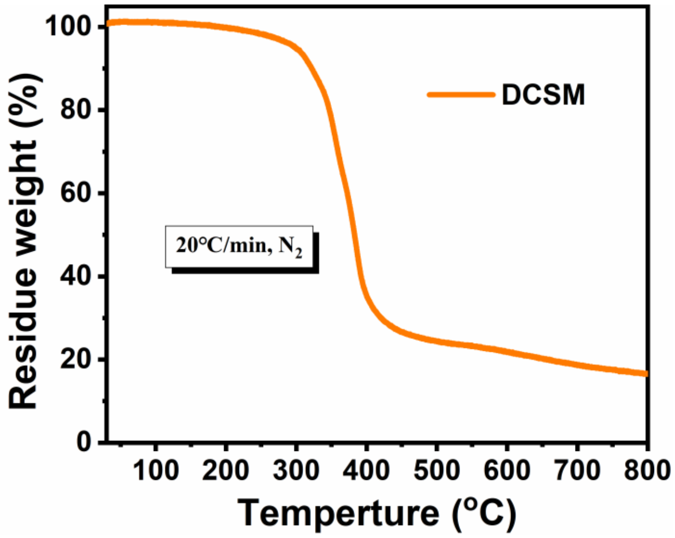


**Supplementary Fig. 16**. The TGA curves of DCSM from room temperature to 800 °C with a heating rate of 20 °C min$^{-1}$.

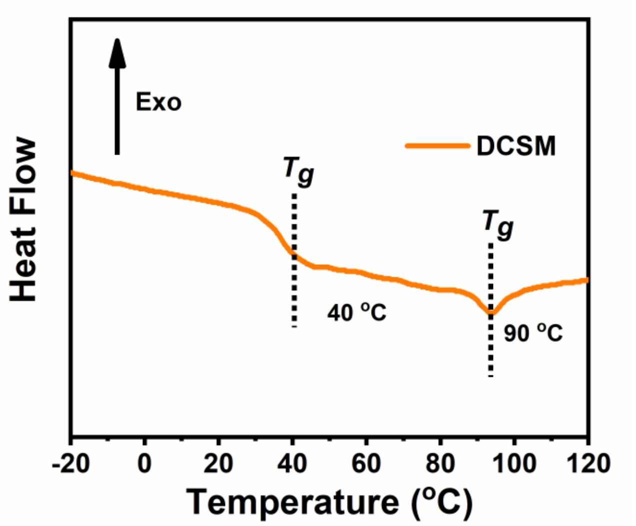


**Supplementary Fig. 17**. The DSC curves of DCSM from -20 °C to 120 °C with a heating rate of 10 °C min$^{-1}$.

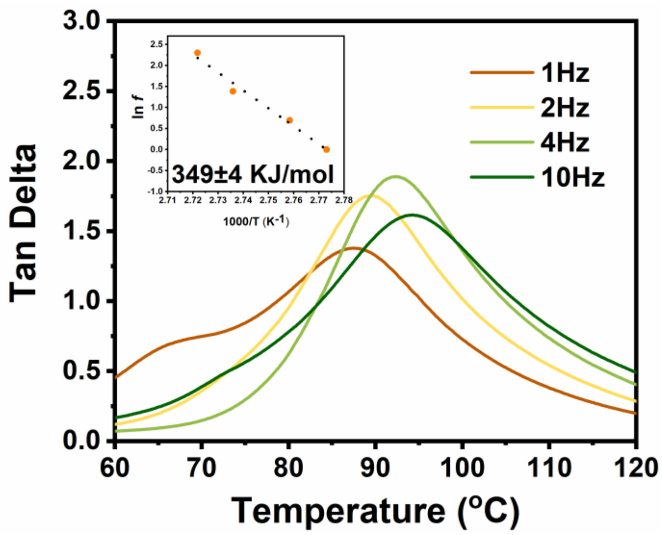


**Supplementary Fig. 18**. Temperature sweeps of DCSM under different frequencies (1 Hz, 2 Hz, 4 Hz, and 10 Hz) and the Arrhenius plot of transitions based on the frequency-dependent shifts of the tan delta peaks (inset).

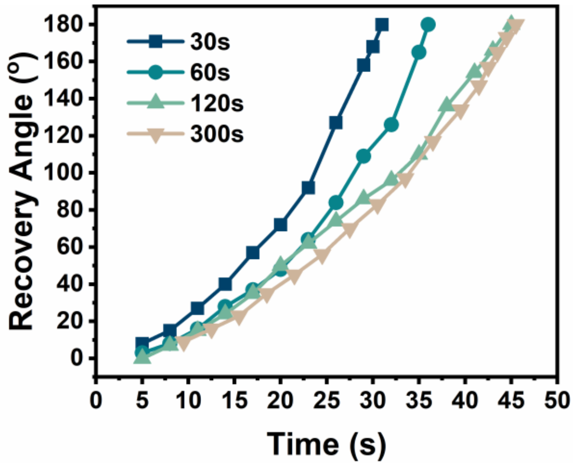


**Supplementary Fig. 19**. Recovery kinetics of DCSM under different programming times.

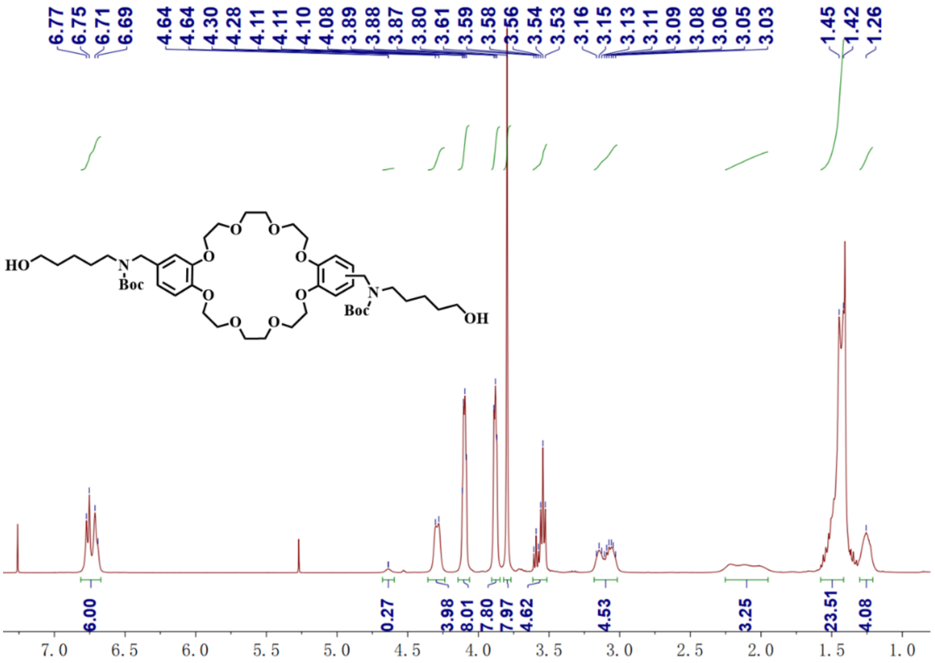


**Supplementary Fig. 20**. The $^{1}$H NMR spectrum of DDCSM-5 (400MHz, 298 K, $CDCl_3$).

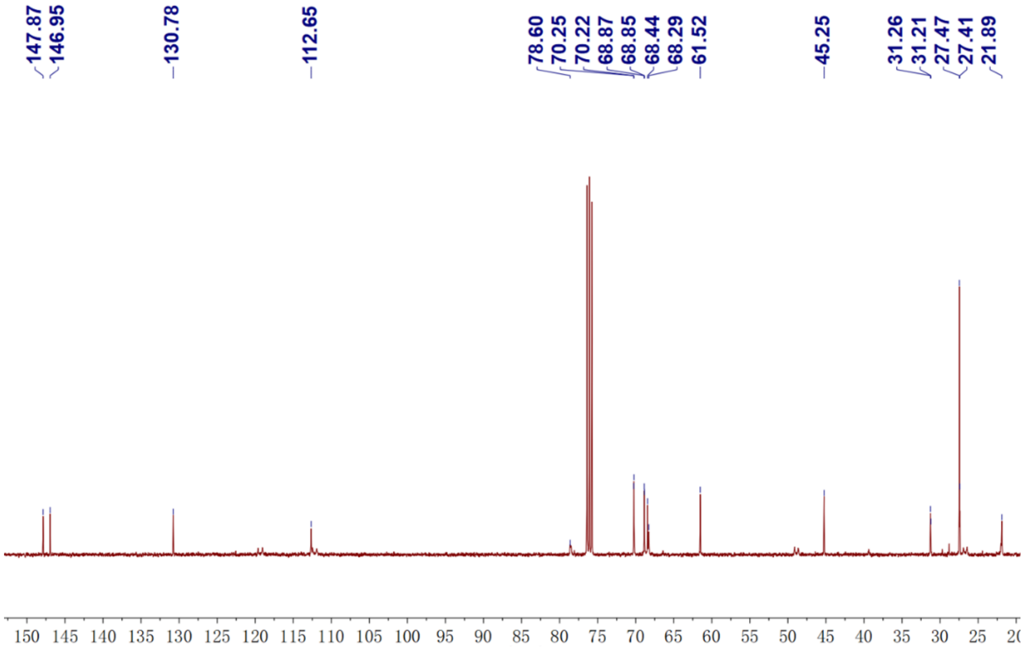


**Supplementary Fig. 21**. The $^{13}$C NMR spectrum of DDCSM-5 (101MHz, 298 K, $CDCl_3$).

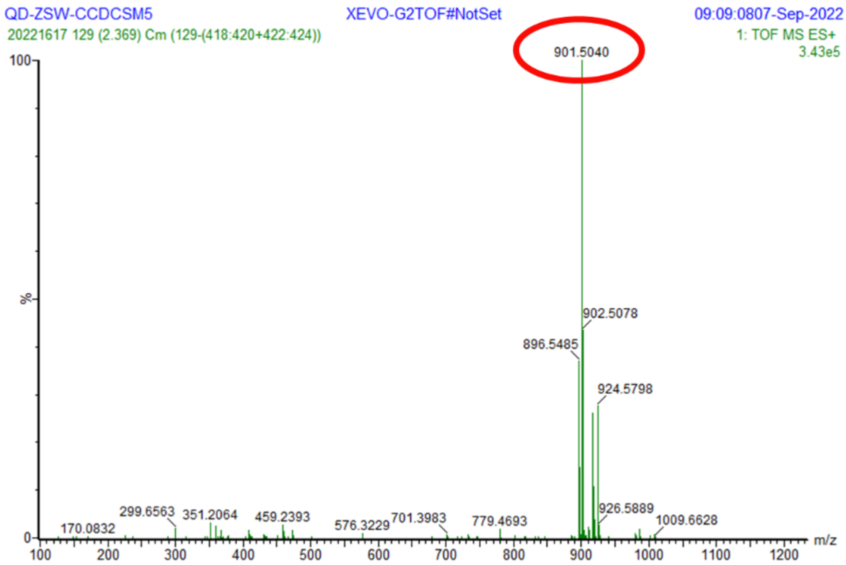


**Supplementary Fig. 22**. The ESI-mass spectrum of DDCSM-5 ([M + $Na^+$] $^+$: 901.5040).

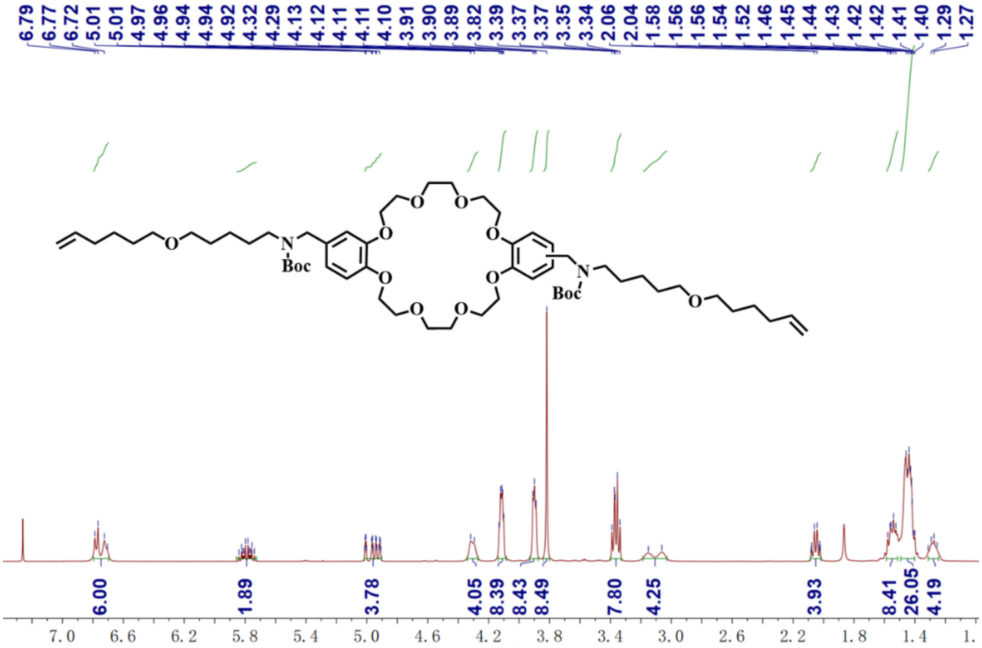


**Supplementary Fig. 23**. The $^1H$ NMR spectrum of DDCSM-6 (400MHz, 298 K, $CDCl_3$).

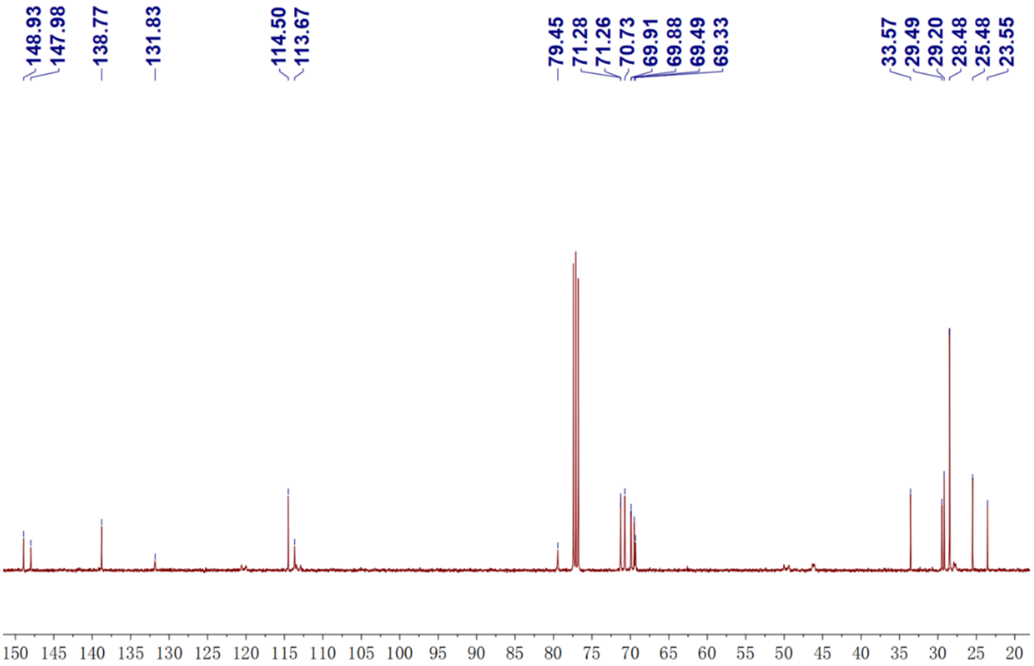


**Supplementary Fig. 24**. The $^{13}C$ NMR spectrum of DDCSM-6 (101MHz, 298 K, $CDCl_3$).

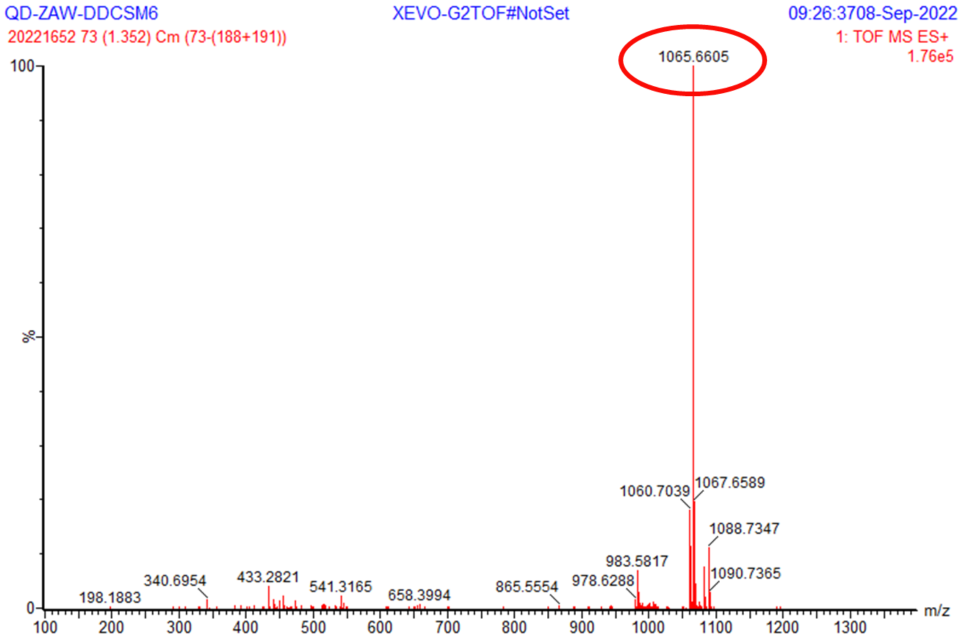


**Supplementary Fig. 25**. The ESI-mass spectrum of DDCSM-6 ([M + Na$^+$] $^+$: 1065.6605).

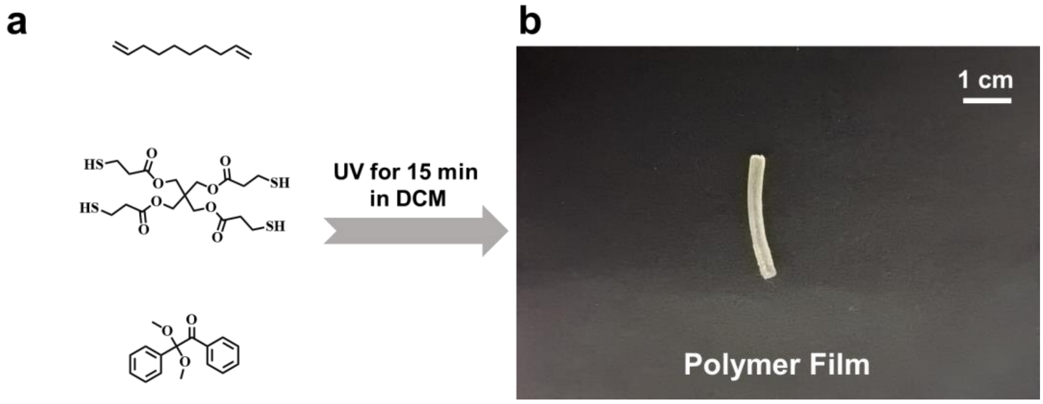


**Supplementary Fig. 26**. **a.** The monomers and condition of preparation for CG-1. **b.** The picture for prepared CG-1 polymer film.

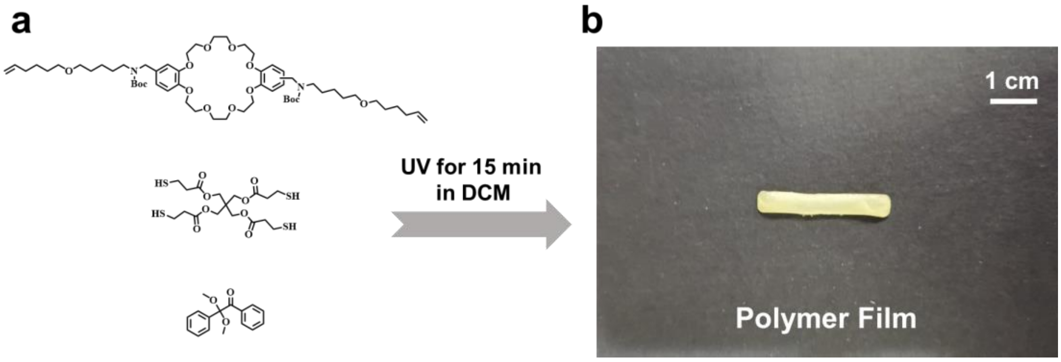


**Supplementary Fig. 27**. **a.** The monomers and condition of preparation for CG-2. **b.** The picture for prepared CG-2 polymer film.

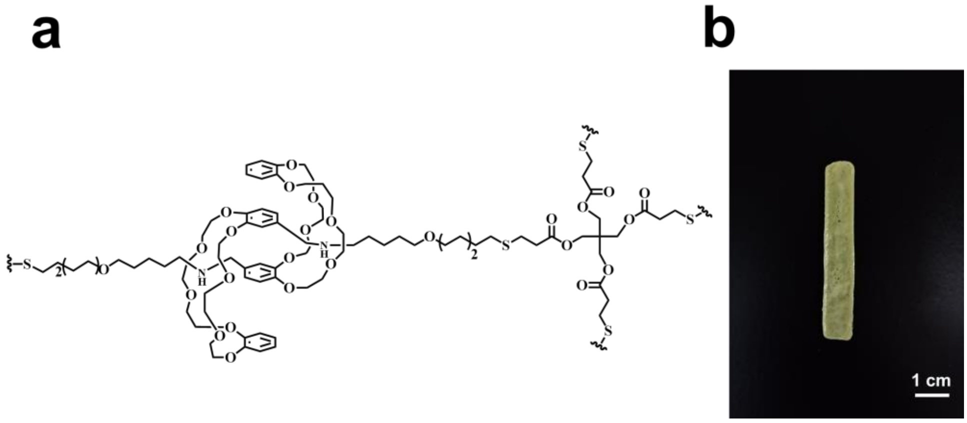


**Supplementary Fig. 28**. **a.** The polymer network structure of CG-3. **b.** The picture for prepared CG-3 polymer film.

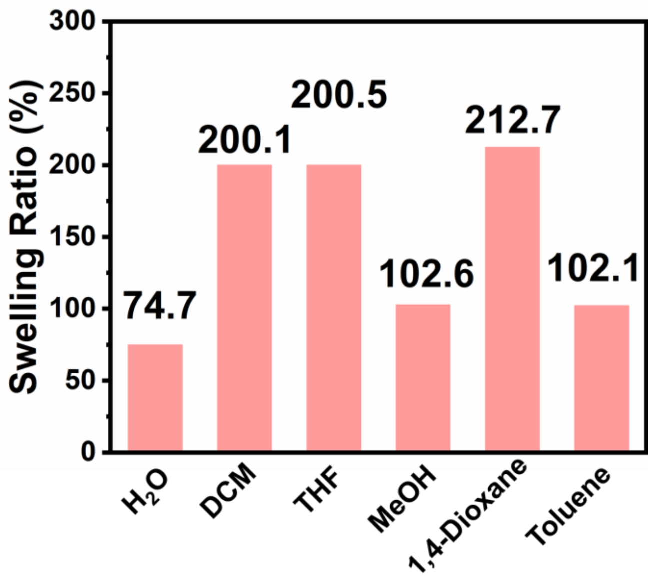


**Supplementary Fig. 29**. The swelling ratio for CG-1 in different solvents for 3 hours.

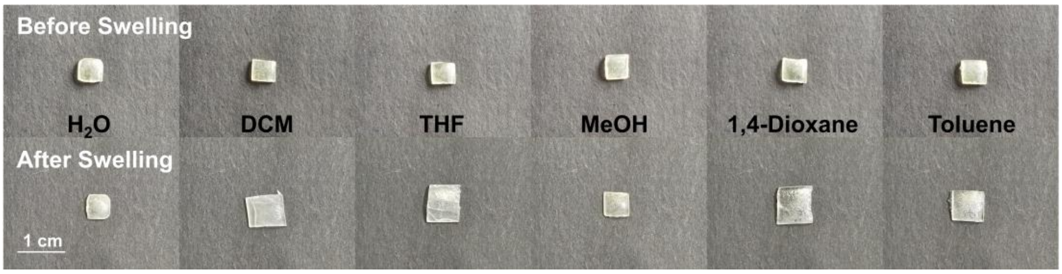


**Supplementary Fig. 30**. The before and after swelling picture for CG-1 in different solvents for 3 hours.

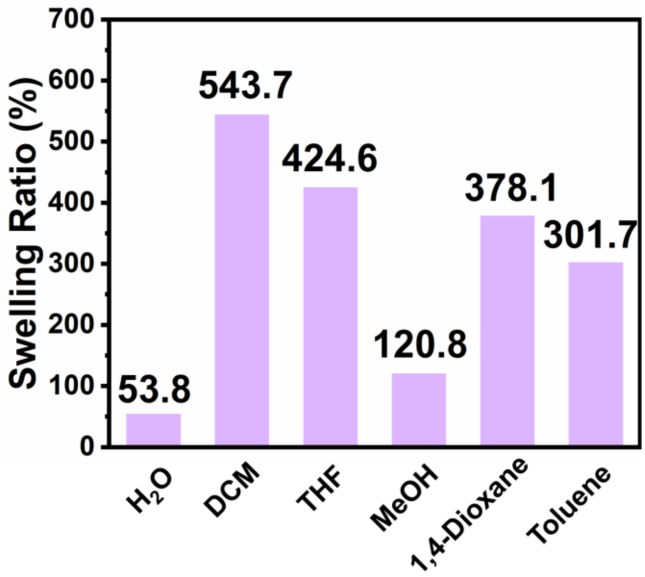


**Supplementary Fig. 31**. The swelling ratio for CG-2 in different solvents for 3 hours.

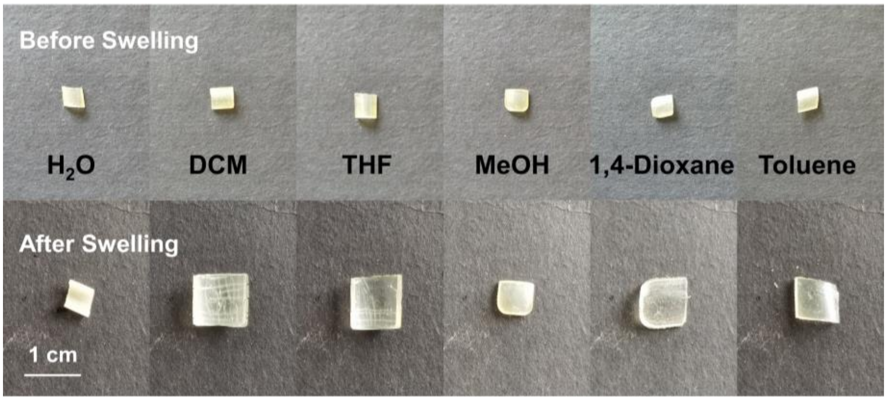


**Supplementary Fig. 32**. The before and after swelling picture for CG-2 in different solvents for 3 hours.

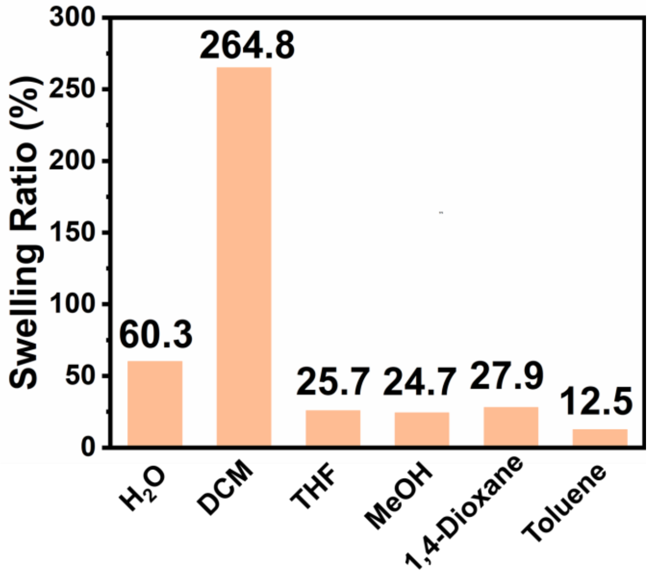


**Supplementary Fig. 33**. The swelling ratio for CG-3 in different solvents for 3 hours.

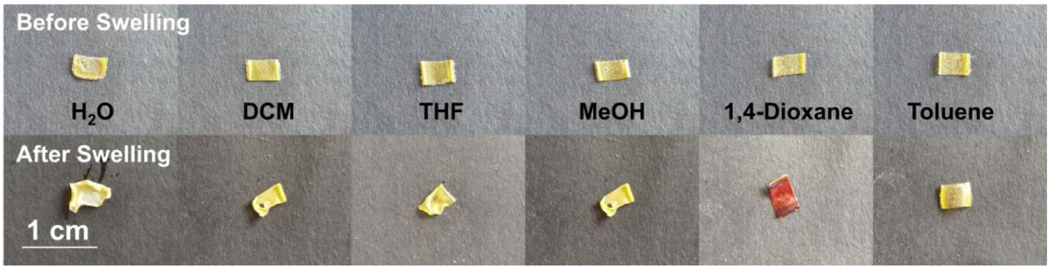


**Supplementary Fig. 34**. The before and after swelling picture for CG-3 in different solvents for 3 hours.

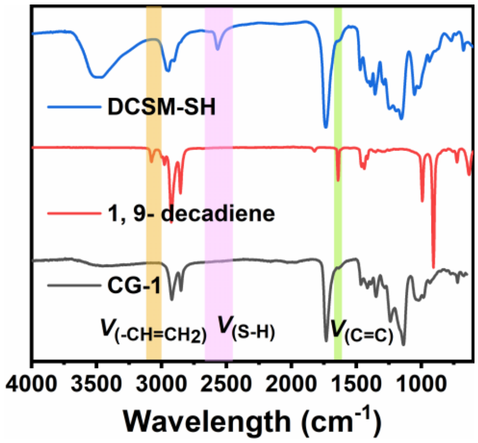


**Supplementary Fig. 35**. The FTIR spectra of CG-1, monomer 1,9- decadiene and monomer DCSM-SH.

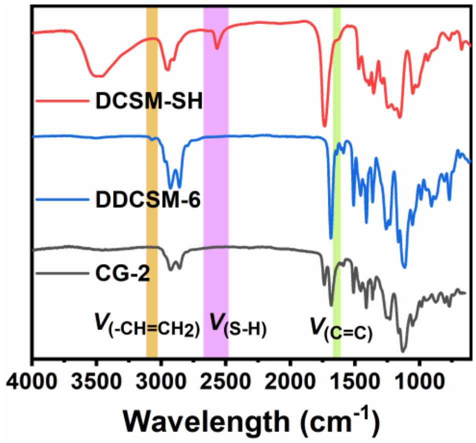


**Supplementary Fig. 36**. The FTIR spectra of CG-2, monomer DCSM-SH and monomer DDCSM-6.

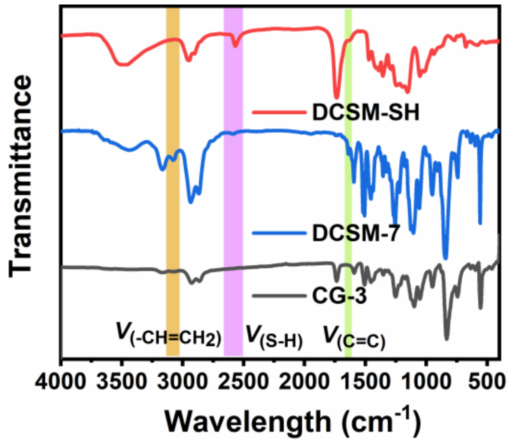


**Supplementary Fig. 37**. The FTIR spectra of CG-3, monomer DCSM-SH and monomer DCSM-7.

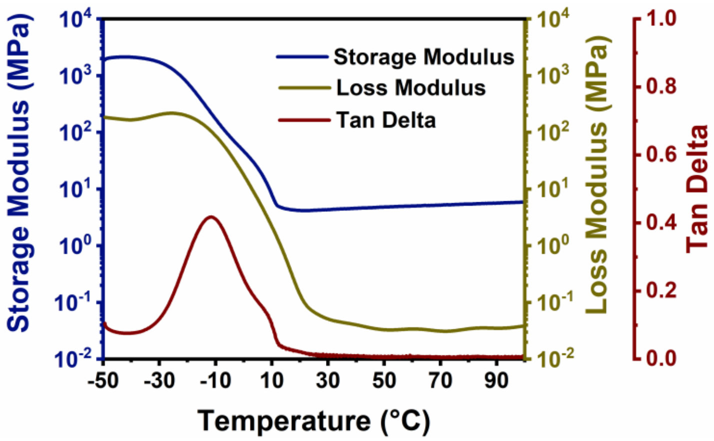


**Supplementary Fig. 38**. The DMA spectra of CG-1 (frequencies: 1Hz).

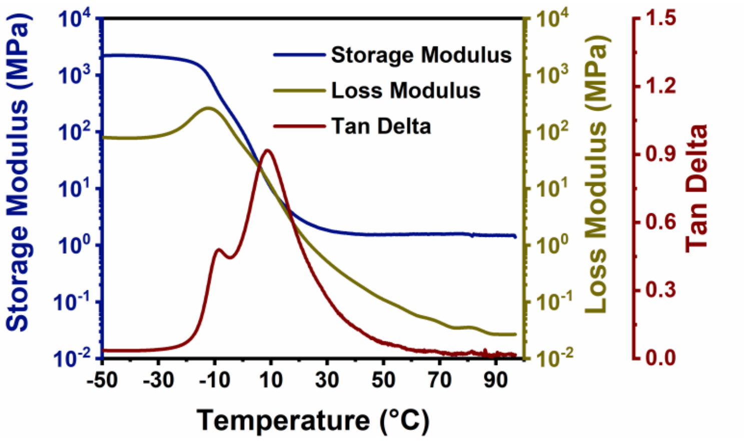


**Supplementary Fig. 39**. The DMA spectra of CG-2 (frequencies: 1 Hz).

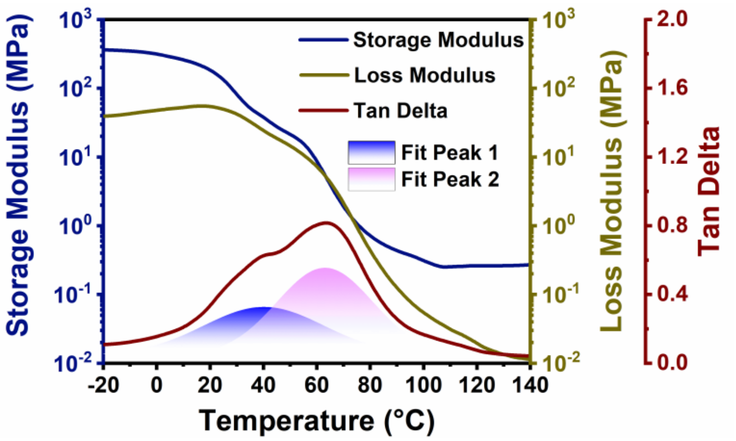


**Supplementary Fig. 40**. The DMA spectra of CG-3 (frequencies: 1 Hz).

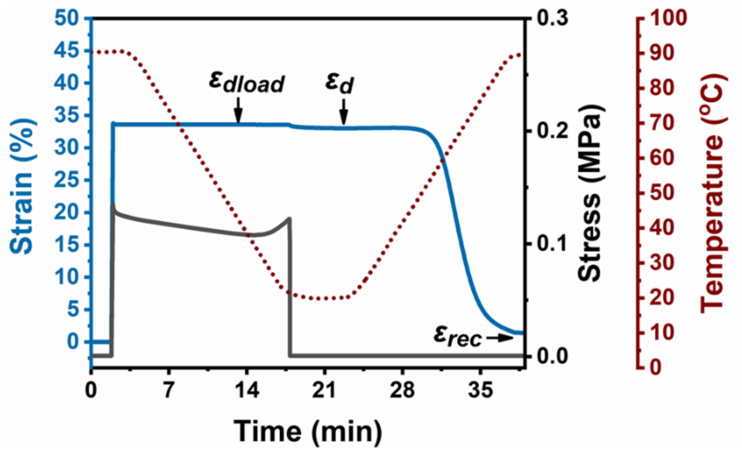


**Supplementary Fig. 41**. The shape memory cycle of CG-3. The $R_f$=97.6% and $R_r$=85.3%. ($R_f$=($\varepsilon_d$/$\varepsilon_{dload}$)*100% and $R_r$=($\varepsilon_d$-$\varepsilon_{rec}$/$\varepsilon_d$)*100%, with $\varepsilon_{dload}$, $\varepsilon_d$, and $\varepsilon_{rec}$ being the maximum strain under load, the fixed strain, and the recovered strain, respectively.

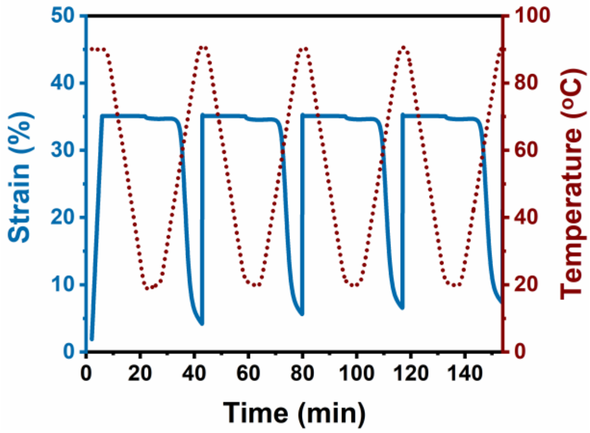


**Supplementary Fig. 42**. The cyclic actuation of CG-3 programmed by uniaxial stretching.

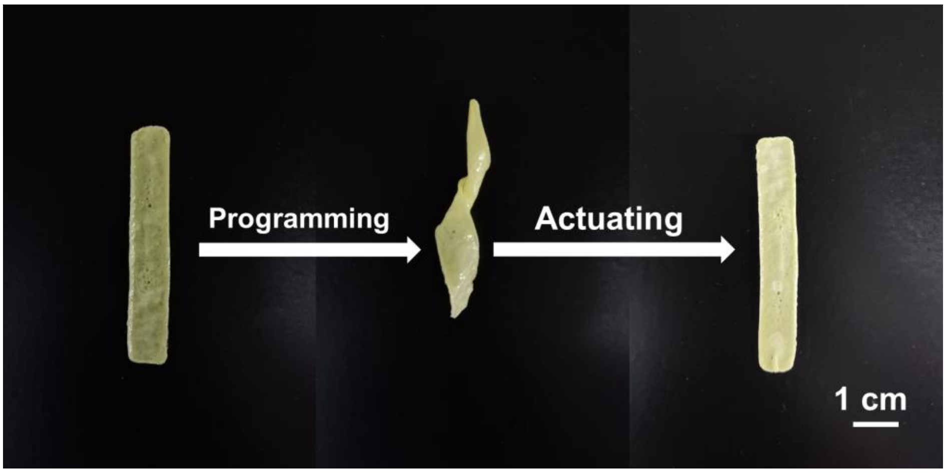


**Supplementary Fig. 43**. The shape-memory photos of CG-3. The initial film is programmed into helical shape at 90 °C, then the programmed shape is actuated to initial shape by heat 90 °C.

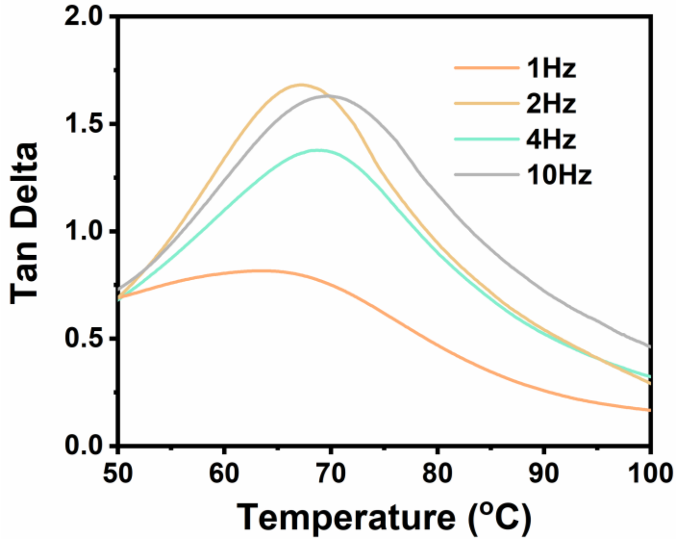


**Supplementary Fig. 44.** The temperature sweeps of CG-3 under different frequencies (1Hz, 2Hz, 4Hz and 10Hz) using DMA.

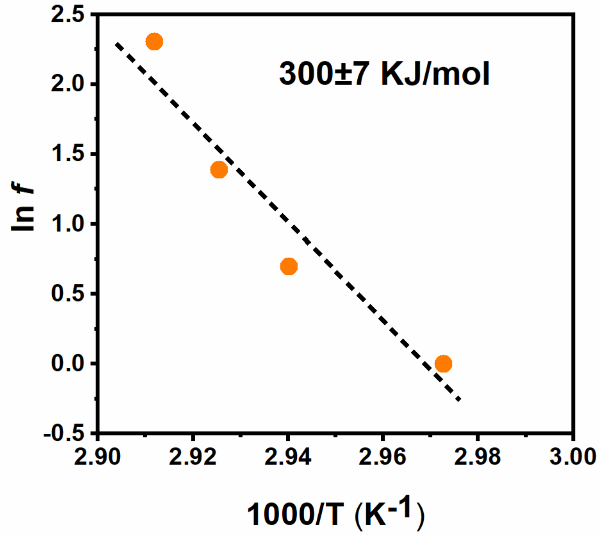


**Supplementary Fig. 45.** The CG-3 Arrhenius plots of transitions based on the frequency-dependent shifts of the tan delta peaks.

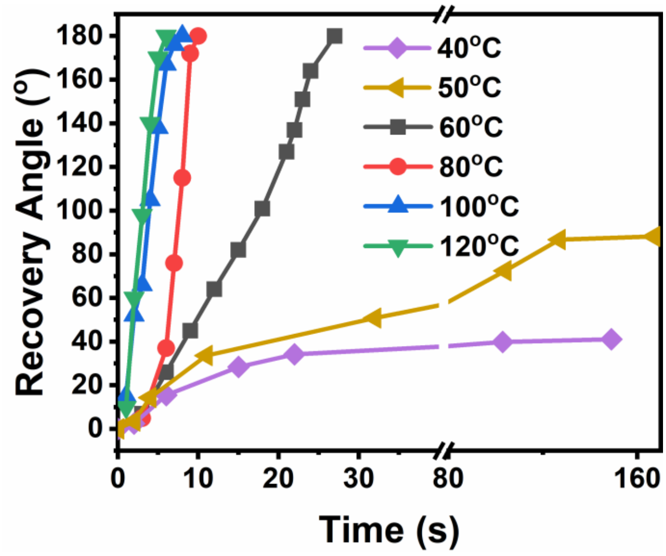


**Supplementary Fig. 46**. Recovery kinetics of CG-3 under different actuation temperatures.

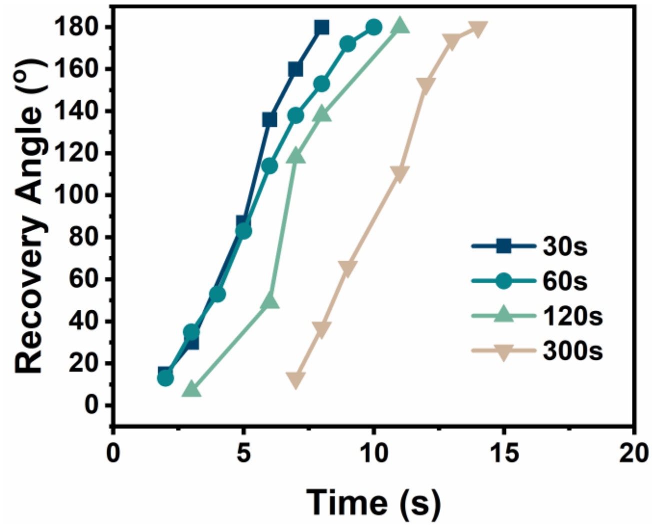


**Supplementary Fig. 47**. Recovery kinetics of CG-3 under different programming times.

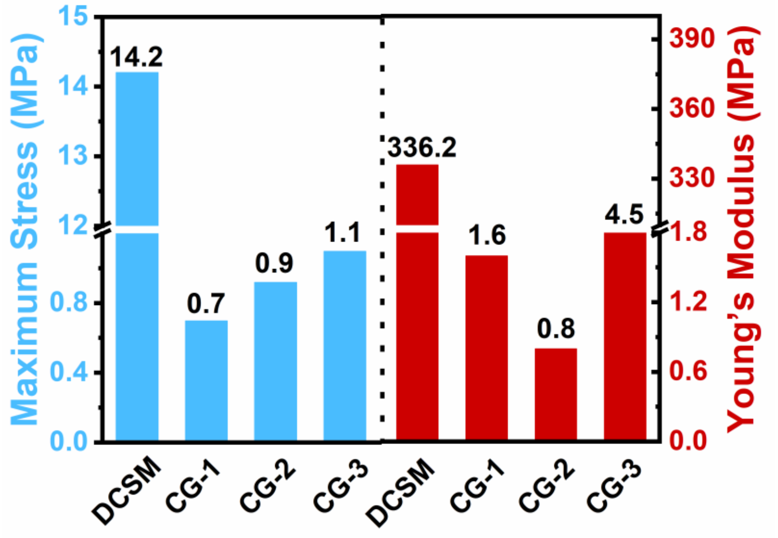


**Supplementary Fig. 48**. The maximum stress and Young's modulus for DCSM and three control groups.

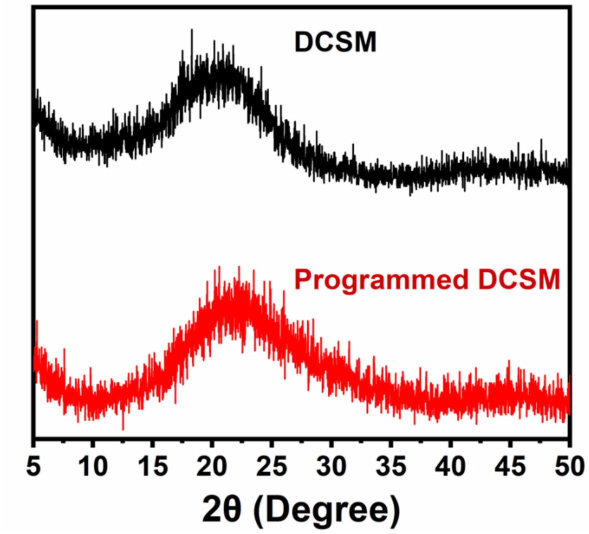


**Supplementary Fig. 49**. The XRD spectra for initial DCSM and programmed DCSM.

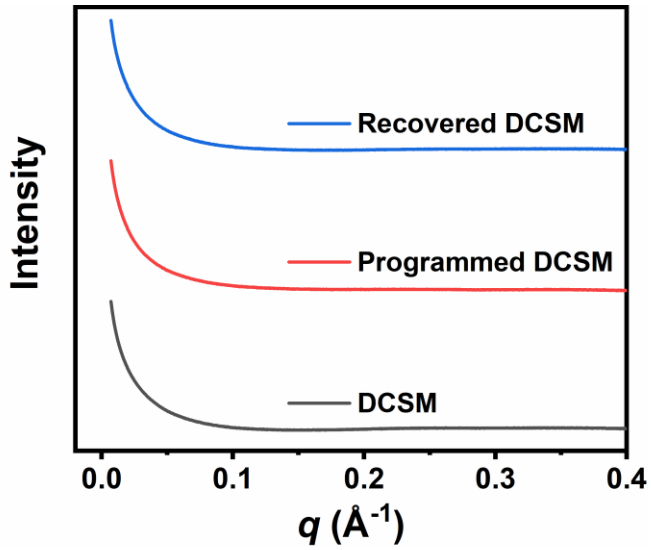


**Supplementary Fig. 50**. The SAXS spectra for initial DCSM, programmed DCSM and recovered DCSM.

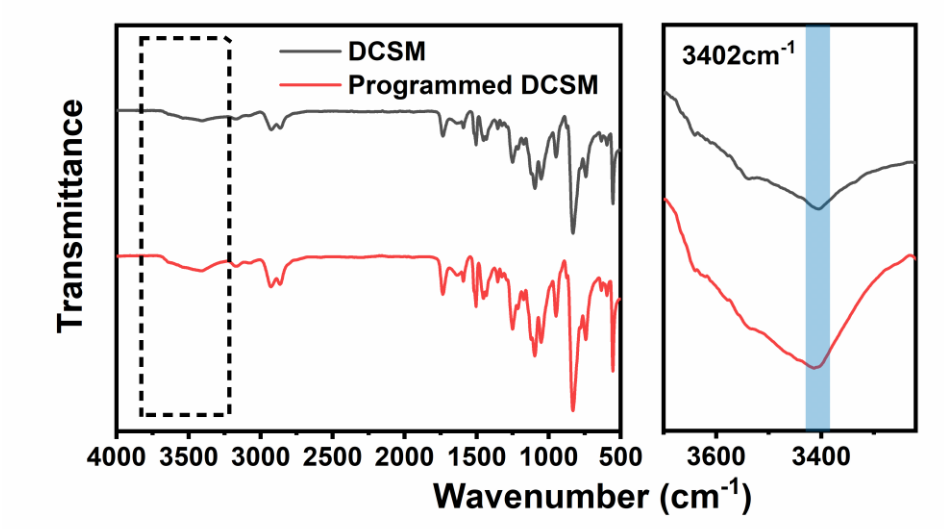


**Supplementary Fig. 51**. The FTIR spectra of DCSM and programmed DCSM. The peak of 3402 $cm^{-1}$ belongs to v(N-H).

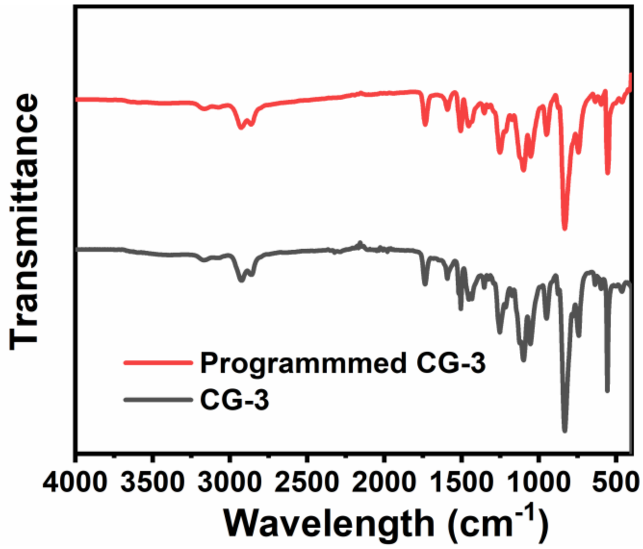


**Supplementary Fig. 52**. The FTIR spectra of CG-3 and programmed CG-3**.**

## 3. Supplementary References

1 Wu, J. et al. An acid-base-controllable c2 daisy chain. Angew. Chem. Int. Ed. **47**, 7470-7474 (2008).

2 Wolf, A. et al. pH and light-controlled self-assembly of bistable c2 daisy chain rotaxanes. Chem. Commun. **51**, 4212-4215 (2015).

3 Fu, X., Zhang, Q., Rao, S.-J., Qu, D.-H. & Tian, H. One-pot synthesis of a c2 daisy-chain-containing hetero 4 rotaxane via a self-sorting strategy. Chem. Sci. **7**, 1696-1701 (2016).

4 Bora, S. J., Dutta, R., Kalita, D. J. & Chetia, B. Novel Isophthalohydrazide-cDB24C8 cryptand derivative for the selective recognition of fluoride ion: An experimental and DFT study. Spectrochim. Acta, Part A **204**, 225-231 (2018).

5 Xu, H. et al. Fluorescence emission enhancement of a T-shaped benzimidazole with a mechanically-interlocked 'suit'. Chem. Commun. **57**, 3239-3242 (2021).
