# Supplementary material for: Mechanically interlocked [c2]daisy chain backbone enabling advanced shape-memory polymeric materials
Source: Nat Commun. 2024 Feb 24;15:1690. doi: 10.1038/s41467-024-45980-y (PMC10894290; doi:10.1038/s41467-024-45980-y)
Supplement: Supplementary file 3 — Description of Additional Supplementary Files [file 41467_2024_45980_MOESM3_ESM.pdf]

Description of Additional Supplementary Files

File Name: Supplementary Movie 1
Description: A DCSM strip film repeatedly programmed into different shapes: letter “E”, “C”, “U”, “S”, “T”. After a polymer film with a permanent shape of “l” was programmed into various temporary shapes as the letters “E”, “C”, “U”, “S”, and “T” at a 90 °C heating plate, it undergoes the recovery processes to the initial “l” shape.

File Name: Supplementary Movie 2
Description: Shape memory behavior of a helical shaped DCSM material. After a helical film of DCSM was programmed into a flat strip at a 90 °C heating plate, it was actuated back to the initial helical state.

File Name: Supplementary Movie 3
Description: Shape memory behavior of a curly shaped DCSM material. After a curly film was programmed into a flat strip at a 90 °C heating plate, it was actuated back to the initial curly state.

File Name: Supplementary Movie 4
Description: Releasing of a stirrer by hot wind. A strip of DCSM was programmed to wrap around a small magnetic stirrer. Upon heating in air by a heat gun, it released the stirrer.

File Name: Supplementary Movie 5
Description: Releasing of a stirrer in hot water. A strip of DCSM was programmed to wrap around a small magnetic stirrer. Upon heating in hot water, it released the object.

File Name: Supplementary Movie 6
Description: Lifting of two paper clips by hot wind. An initial helical DCSM film hanging two clips was programmed into flat to allow the lifting of the clips after heating in air by a heat gun.

File Name: Supplementary Movie 7
Description: Lifting of two paper clips in hot water. An initial helical DCSM film hanging two clips was programmed into flat to allow the lifting of the clips after being heated in hot water.
